# Therapeutic Improvement in People With Schizophrenia Undergoing tACS/CBTp (Transcranial Alternating Current Stimulation/Cognitive Behavioral Therapy for Psychosis) Associated With Usual Medication Regimen: Protocol for a Pilot, Randomized, Single-Blind Trial

**DOI:** 10.2196/80593

**Published:** 2026-02-10

**Authors:** Felicia Iftene, Adriana Farcas, Simon O'Brien, Christopher Bowie, Michael Best, Oyedeji Ayonrinde, Terry Landry, Jennifer Carlson, Scott John Davidson, Ellie Rodgerson, Ashley Theis

**Affiliations:** 1 Division of Adult Psychoses Department of Psychiatry Queen's University Kingston, ON Canada; 2 Providence Care Mental Health Services Kingston, ON Canada; 3 Department of Psychology Queen's University Kingston, ON Canada; 4 Department of Psychology University of Toronto Toronto, ON Canada; 5 Center for Neuroscience Studies Queen's University Kingston, ON Canada; 6 Research Compliance and Training- Vice-Principal Research Portfolio Queens University Kingston, ON Canada

**Keywords:** schizophrenia, tACS, transcranial alternating current stimulation, CBTp, cognitive behavioral therapy for psychosis, EEG, electroencephalogram, heart rate variability, gender, clinical or functional improvement

## Abstract

**Background:**

Transcranial alternating current stimulation (tACS) applies a low-intensity sinusoidal electrical current through electrodes placed on the scalp to boost the brain’s own oscillation by way of entrainment. When a single frequency is applied, this exogenous oscillation synchronizes with the brain’s endogenous frequency. Gamma frequency synchrony stands out as a binding mechanism for integrating disparate brain networks, mediating perception, cognition, and memory, typically disturbed in schizophrenia. The treatment of schizophrenia includes medication and cognitive behavioral therapy for psychosis (CBTp). We are adding tACS to these usual treatments, targeting gamma oscillation stimulation, to augment the CBTp efficacy in people living with schizophrenia.

**Objective:**

This study aims to elicit cognitive readiness and therapeutic engagement by adding tACS to each CBTp session in individuals with schizophrenia taking their usual medication, to evaluate the possible improvement of the level of functioning, and to determine if the response to intervention is gender specific.

**Methods:**

This is a pilot, prospective, randomized, repeated-measures, single-blind study design. We expect to enroll 28 participants randomly assigned to one of two treatment arms: arm 1 (tACS/CBTp, n=14) or arm 2 (sham tACS/CBTp, n=14; tACS is sham, but CBTp is active). The intervention with active or sham tACS/CBTp will take place weekly for 16 weeks. Primary outcome measures—electroencephalogram, Positive and Negative Syndrome Scale, 16-item Negative Symptom Assessment, and Cognitive Flexibility Scale—will evaluate the efficacy of treatment at the end of the intervention and at the two follow-ups. We will use SPSS (version 29, IBM Corp); the main tests will include repeated measures and mixed design ANOVA.

**Results:**

The timeline for recruitment, treatment, and follow-ups is 18 months, followed by 6 months for data analysis, writing manuscripts, and dissemination activities. By November 1, 2025, we have enrolled 15 participants: 12 are following the intervention protocol (8 active and 4 sham tACS). Two participants were screening failures, and one participant withdrew after intervention 2.

**Conclusions:**

Our expectations are as follows. CBTp will improve the scores of psychological and psychosocial tests at the end of therapy for both groups, but it will be superior for the group with tACS intervention. Considering that cognitive and emotional status is gender dependent (hormonal differences, brain structure, and sociocultural influences), we expect that the therapeutic response could be gender specific. CBTp will enhance electroencephalogram activity and the heart in clients with schizophrenia at the end of therapy for both groups, but it will be superior for the group with tACS preintervention. The baseline heart rate variability will predict symptom improvement and will increase over the course of therapy. We hope our research will improve the treatment of people living with schizophrenia, thereby enhancing their quality of life, reducing the rate of relapse, and lowering the costs of care.

**Trial Registration:**

ClinicalTrials.gov NCT06889025; https://clinicaltrials.gov/study/NCT06889025

**International Registered Report Identifier (IRRID):**

PRR1-10.2196/80593

## Introduction

### Background and Scientific Rationale

Schizophrenia is a debilitating neurodevelopmental disorder with tremendous consequences for the individual and family, resulting in an important socioeconomic burden. There are approximately 360,000 people affected by schizophrenia in Canada. The illness usually begins between the ages of 15 and 35 years, during key developmental milestones of life. Ontario has the largest number of individuals living with schizophrenia; 84,000 Ontarians (and their families) are affected by treatment-resistant schizophrenia.

The mainstay treatment of schizophrenia is antipsychotic medication. However, antipsychotics do not work adequately for up to 60% of individuals [1]. Although evidence shows that medication has beneficial effects on positive symptoms (hallucinations, delusions, etc) and reduces relapses, a significant number of patients do not fully respond to it [2]. The persistence of symptoms throughout its evolution, compounded by the added side effects of medication, leads to significant impairments in social and relational aspects of life, contributing to the significant distress and disability associated with the disorder. Combined, early medical and psychosocial interventions are consistently sought, while research explores new avenues to enhance current approaches or attempt stand-alone, successful, cost-effective, and noninvasive treatments [3]. When cognitive behavioral therapy for psychosis (CBTp) is used alongside medication, some improvements have been observed. Still, the debilitating negative symptoms remain (such as reduction of emotional expression, lack of social involvement, inability to initiate goal-directed activities, etc), which are key factors in long-term disability [4,5].

Schizophrenia is characterized by abnormalities in neural circuitries resulting in dysfunctional cognition and behavior [6,7]. A new treatment, transcranial alternating current stimulation (tACS), has therapeutic results in Alzheimer disease [8-10]. Recent randomized double-blind clinical trials of tACS in Alzheimer disease showed a significant improvement in memory performance, along with restoration of intracortical connectivity, as compared to sham tACS [11-13]. Recent systematic reviews discuss the general optimism within the scientific community regarding the use of electrical currents in enhancing cognitive performance in healthy people and ameliorating symptoms in a wide variety of conditions, including neuropsychiatric ones [6,14-16]. The rationale behind such an approach rests on the understanding that neural oscillations are a fundamental mechanism that organizes the temporal relationship of activity patterns in brain networks [2,17,18]. Certain psychiatric disorders were identified over the last decades as showing imbalances in network connectivity, showing high or slow oscillatory activity by comparison with the healthy population. If tACS improves memory in Alzheimer disease by modulating brain oscillations, then it may also enhance cognitive function in schizophrenia by targeting similar neural mechanisms, particularly those involving working memory and gamma oscillations. Of note, gamma synchrony stands out as a binding mechanism for integrating disparate brain networks, mediating perception, cognition, and memory [3], which is typically disturbed in schizophrenia. Manifesting as synchronous high-frequency oscillations of brain electrical activity, it occurs across several brain regions whose functions are integrated this way [19,20]. Global delays and decreased temporal connectivity in neural activity stemming from impairments in frontal lobe synchrony may be relevant to the disordered cognitive control and flexibility in schizophrenia [21-23].

tACS, the focus of our study, applies a low-intensity sinusoidal electrical current to the brain through electrodes placed on the scalp, and it is thought to boost the brain’s own oscillation by way of entrainment. When a single frequency is applied, this exogenous oscillation will synchronize with the brain’s endogenous frequency. Conversely, multiple frequencies lead to desynchronization of cortical oscillations [24]. The value of the frequency applied thus becomes a determining factor in the effects sought and identified in tACS studies.

Research focusing on tACS mechanisms has shown that effects result from manipulating the membrane potential of neurons that are aligned with the introduced electric field, mostly pyramidal cells in layer V [25,26]. Upon stimulation by tACS, which will cause an alternating change in membrane potential, these cells demonstrate a resonance specific to the frequency of stimulation, with long-term aftereffects—70 minutes after one stimulation session for 20 minutes [19]—and implications on oscillatory cortical connectivity between different cortical regions [27]. In auditory hallucinations, research has demonstrated interhemispheric miscommunication, where tACS can manipulate auditory perception in healthy participants by decoupling this interhemispheric connectivity [28]. The advantage of using tACS over other noninvasive brain stimulation modalities lies in its effectiveness in entraining endogenous brain oscillations, as it mimics the alternating nature of these oscillations [29,30].

From a practical standpoint, tACS exhibits superior cost, portability, tolerability, and safety profiles [17]. tACS is a feasible tool that reshapes or resynchronizes intrinsic brain rhythms, manipulating the associated brain functions without adding extra excitatory or inhibitory burden.

We hypothesize that tACS targeting gamma oscillations, added to each CBTp session, will improve cognitive performance in individuals living with schizophrenia under their usual antipsychotic medication by restoring neural synchrony and enhancing cortical connectivity. This will subsequently improve clinical and functional status and medication adherence.

### Proposed Therapeutic Protocol

The investigators are proposing a new, noninvasive therapeutic model using tACS to augment CBTp efficacy in individuals with schizophrenia. By selecting electroencephalogram (EEG) brain oscillation activity as a biomarker for the progression of cognitive deficits in schizophrenia, this research seeks to determine whether addressing oscillation perturbations can mitigate cognitive deficits. The heart rate variability (HRV) was selected as a biomarker of improvement of somatic and mental health. The investigators are also aiming for an analysis through a Gender-Based Analysis Plus lens by using the Bem Sex-Role Inventory (BSRI), along with specific tests for psychosis (Positive and Negative Syndrome Scale [PANSS], 16-item Negative Symptom Assessment [NSA-16], etc).

### Objectives

This study compares the effects of a harmless, low-voltage electrical stimulation (tACS) with placebo stimulation (sham tACS) applied for 20 minutes at the beginning of the CBTp session to increase the efficacy of psychotherapy in people living with schizophrenia.

Primary objectives are to determine whether (1) the new intervention protocol using tACS will enhance the EEG activity, expecting that CBTp will enhance EEG activity in clients with schizophrenia at the end of therapy for both groups, with a better outcome for the group with active tACS stimulation; and (2) tACS applied at the beginning of the CBTp sessions in people with schizophrenia will predict better responses to therapy (compared with the sham tACS/CBTp). CBTp is expected to improve the scores of the PANSS, NSA-16, and Cognitive Flexibility Scale (CFS).

The secondary objectives are to determine whether (1) there will be an improvement of level of functioning and quality of life in both intervention groups, with a superior outcome for the group receiving tACS intervention (psychological and psychosocial tests); (2) higher in-session HRV predicts a better response to CBTp (the expectation is that baseline HRV will predict symptom improvement and increase over the course of therapy); and (3) the response to tACS/CBTp intervention is gender specific; considering that cognitive and emotional status is gender dependent, we expect that the therapeutic response could be gender specific.

## Methods

### Trial Design

#### Description

**Figure 1:**
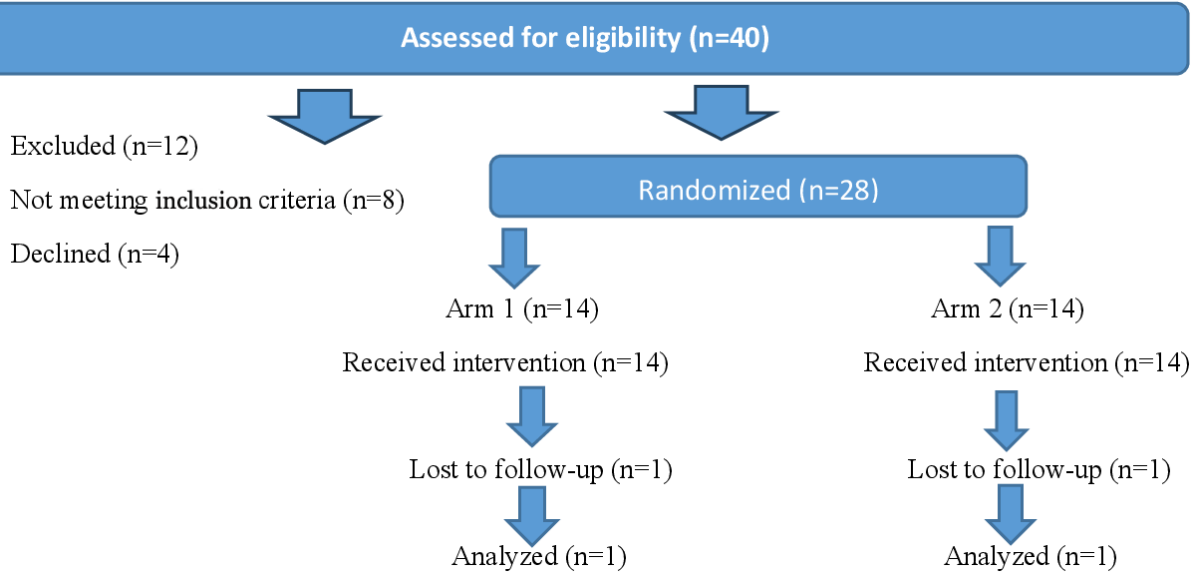
CONSORT (Consolidated Standards of Reporting Trials) flow diagram.

This is a prospective, randomized, repeated-measures, single-blind study design. Preintervention, eligible participants will be randomly assigned to one of two treatment arms: arm 1 (tACS/CBTp, n=14) or arm 2 (sham tACS/CBTp, n=14; tACS is sham, but CBTp is active). In the intervention (16 weeks), participants in arms 1 and 2 will receive once-weekly tACS/CBTp or sham tACS/CBTp. Individuals with schizophrenia (*DSM-5* [*Diagnostic and Statistical Manual for Mental Disorders* {Fifth Edition} criteria) stratified by age and sex. The investigators expect 150 potentially eligible patients from Providence Care Mental Health Services, with 33-40 participants consenting to participate and 28 participants undergoing the research activities (see Figure 1).

Participants will be randomized 1:1 to one of two arms and stratified by age (18-30, 31-55, and >60 years) and by sex (male or female).

#### CBTp Intervention

Participants on both arms will receive CBTp in 16 individual-based, 50-minute-long, weekly sessions with 2 booster CBTp sessions (with active or sham tACS) at 1-month and 3-month follow-up visits (after intervention).

#### tACS/Sham tACS

Participants randomized to the tACS arm will receive gamma band electric stimulation for 20 minutes at the beginning of each CBTp session. After the first 20 minutes of brain stimulation, the tACS device automatically stops, and the device remains in place while the CBTp session continues to run for 30 more minutes.

Participants randomized to the sham tACS arm will receive sham tACS for 20 minutes at the beginning of each CBTp session. After the first 20 minutes of sham tACS, the device automatically stops, and it remains in place, while the CBTp session continues to run for 30 more minutes.

### Selection and Withdrawal of Participants

#### Inclusion and Exclusion Criteria

The inclusion and exclusion criteria for the study participants are listed in Textbox 1.

Changes in medication/new hospitalization for worsening symptoms and/or presence of suicidal ideation are no more exclusion criteria for participants that have passed visit 5 (end of intervention, during follow-up stage); however, the results of the follow-up visits will count at the final results, depending on the severity/imminent danger of symptoms and medication changes, at the best judgment of the principal investigator (FI).

**Inclusion and exclusion criteria.**

**Inclusion criteria**

- Individuals, with at least 5 years’ duration of illness, who meet diagnostic criteria for schizophrenia according to the *Diagnostic and Statistical Manual for Mental Disorders* (Fifth Edition) and at least one residual positive symptom (as determined by the referring physician)
- No change in medication regimen for at least 1 month, preferably 3 months (minor dose adjustments and/or change in medication involving symptoms, such as sleep, anxiety, or medical symptoms such as fever and pain, are permitted)
- All genders between ages 18-65 years; participants >65 years may be eligible if cognition assessed by the Montreal Cognitive Assessment score is greater than or equal to 26
- Ability to understand English with a reading level at or above grade 6
- Able to understand and comply with the requirements of the study
- Provision of written informed consent

**Exclusion criteria**

- Current drug substance use
- Current suicidal ideation/plan
- Current enrollment in cognitive behavioral therapy for psychosis or other formalized psychosocial interventions
- Undergone vagotomy or surgery on the vagus nerve
- Comorbid neurological condition, including seizures
- Current fibrillation or pacemaker
- Severe or moderate intellectual disability
- Currently undergoing hormone therapy
- Under 18 years of age
- Changes in medication/new hospitalization for worsening symptoms and/or presence of suicidal ideation

#### Criteria for Withdrawal

Premature withdrawal or suspension may occur if imminent safety concerns are disclosed to a study team member or a member of the CBTp Clinic by the participant. Withdrawal would be considered with the best interests and safety of the participant considered foremost. An example of this would be if a participant indicated suicidal or homicidal ideation with a plan to carry out such an act at any point in time. After safety considerations are met, the participant would be eligible to return or be included in the study. The research team may temporarily or permanently halt the study if there is any reason to believe that a participant has a medical reason to discontinue participation. Some of the reasons why this might happen are listed below:

- Their condition worsens or does not improve, and their doctor thinks they need a different treatment.
- The study treatment or procedures are found to be unsafe or ineffective.
- They are unable to follow instructions given to them about the study, or they otherwise cannot do or continue to do what they need to participate in the study.
- They develop another serious disease.
- They become pregnant.
- Cancellation by the sponsor or for other unforeseen reasons that make it necessary to stop their study participation.

If a participant is removed from the study, the study doctor will explain to them why the participant was removed. A participant may also be temporarily suspended or stop their participation in the study if they require attention due to a risk of harm to themselves or others. This may be indicated by a member in their circle of care or by the clinician-researchers on the team. In the event there is a risk of harm to themselves or others, after the risk is no longer deemed significant, they may be able to continue the study.

If participants who have passed visit 5 (end of intervention, during the follow-up stage) and do not withdraw their consent to participate, their data can be considered for use.

Participant withdrawal forms have been created to accurately document any withdrawal.

#### Inclusion of Women and Minorities

Our project is gender and minority inclusive. All participants included will have a diagnosed mental health disorder of schizophrenia. The investigators plan to include individuals who are both inpatients and outpatients at Providence Care Hospital. This includes participants who may be in long-term care.

#### Disability

Individuals will be excluded if they have severe or moderate intellectual disability or dual diagnosis due to the nature of CBTp, which requires the learning of coping strategies, adequate attention and memory, engagement using speech, and appreciation of consequences of action, thoughts, behaviors, emotions, and beliefs, which are limited in severe intellectual disability.

#### Language and Linguistic Proficiency

Individuals will be excluded if they cannot understand English with a reading level at or above grade 6. The ability for an individual to meaningfully engage in a CBTp talk therapy would be limited if their English level is limited to a grade 6 level and is not thought to be substantially beneficial to the individual.

#### Age

Participants recruited will be included if they are aged 18-65 years. Individuals older than 65 years of age will be required to be assessed by the Montreal Cognitive Assessment (MoCA) and will be included in the study if results validate adequate cognitive performance. The MoCA will be administered at the screening stage (a score under 26 will be the exclusion criterion).

### Recruitment

A letter and a poster will be distributed to psychiatrists at Providence Care Mental Health Services, informing them that they can refer clients to the CBTp Clinic, and they are invited to refer potential participants who meet an inclusion or exclusion criterion. Word of mouth and brochures or flyers will be prepared. The clinic staff or the participant will then contact our research coordinator or assistant to provide the potential participant with more information. The investigators plan to include individuals who are both inpatients and outpatients at Providence Care Hospital. This includes participants who may be in long-term care. Potential participants in the study will be referred to the CBTp Clinic at Providence Care Hospital by the participant’s primary treating psychiatrist.

During the first meeting at the CBTp Clinic, the potential participant will be informed about the study. The potential participant will be informed that their participation in the study is entirely optional, alongside the standard clinic disclosures about privacy/confidentiality. They will have the opportunity to consider further involvement in a study, with a break provided if they choose to meet with clinic personnel and provide written consent.

Express written consent to participate in the study will be obtained by a member of the research team who is not in the circle of care of the participant (at arm’s length).

### Participant Information and Informed Consent Procedures

Before data collection, potential participants will be fully informed of the observational nature of the study, in that the sponsor intends to collect information and follow the course of therapy in clinical practice. They will be told that their consent to allow the collection of information within the context of this noninterventional study is voluntary and may be withdrawn at any time. Only participants who are fully able to understand the nature of the research and provide their consent voluntarily will be enrolled. Participants must provide their informed consent after a comprehensive and understandable explanation of the clinical study’s nature, scope, and potential consequences. After reviewing the document, participants must give written consent. The consent process must be validated by the personally dated signatures of both the participant and the person facilitating the informed consent discussion. Participants will receive a copy of the signed consent document, while the investigator will keep the original. No study-specific procedures will be carried out until valid consent is obtained. If the participant agrees, their primary physician will be informed about their participation in the study. Where a participant has difficulty reading, a member of the research team will verbally communicate the information on a form or questionnaire if a participant requires clarification. Every effort will be made to help eligible participants with communication difficulties. In this study, the investigators do not enroll participants who are incapable of signing informed consent. To proactively monitor changes in the status of capacity, midway through the study, the research team will seek confirmation of the capacity status of the participant from health care personnel. As is typical ethical practice outside the scope of this study, if members of the CBTp Clinic (by way of observation or professional judgment) suspect a change in capacity in the participant, the participant’s primary treating psychiatrist will be notified, and a reassessment will take place.

### Reimbursement

The investigators will provide each participant with a US $10 reimbursement to cover out-of-pocket expenses, such as a snack and bus ticket, for each of the 6 required visits as part of the study. Payments will be provided after each task is complete. Participants will be encouraged to bring a lunch with them, as indicated on the informed consent form.

### Enrollment and Randomization

After the screening visit, participants will be randomized in a 1:1 ratio with stratification according to age and sex.

Maintaining the blind is covered by the device, which induces a discrete tickling sensation at the starting point of the intervention for participants on sham tACS. This study is single blind, being blinded only for participants. In case of emergency, the research team would be aware of the interventional (or sham) nature of the problem. Communication with participants regarding this aspect will be possible only at the end of the study, at the end of visit 6 (second follow-up), at the patient’s request, or after they withdraw from the study. At the study closure, each participant will receive a debriefing letter containing unblinding disclosure.

### Trial Intervention

#### tACS

This trial uses a Soterix Medical 1×1 Mini-CT tES Stimulator, licensed for use in Canada. It is a small and portable device, with 3 rechargeable 800 mAh batteries and the necessary accessories and calibration to begin tACS right out of the box. The device does not allow initiation of stimulation if the contact quality is outside the desired range. If the session is already underway, the device will alert the user with continuous beeping to prompt immediate corrective action. Stimulation is also recorded by the device, which provides a detailed log of the stimulation session, including pause events, critical events such as poor contact quality, and the total time of the session. Frequency has a 1 Hz resolution and can be adjusted from 1 to 200 Hz. Current intensity has a 0.01 mA resolution and can range from +0.10 to 4.00 mA. Session duration and ramp duration are also adjustable, with session times ranging from 5 to 60 minutes and ramp times from 10 to 30 seconds. The electronic unit is intended to generate the electrical pulses of a specified waveform, duration, and repetition rate. The pulses are delivered to a patient via stimulating electrodes positioned at the patient’s head according to the required technique. The mini-CT is European Union Conformity marked. The SNAP Headset can be used to position Soterix Medical SNAP pads at designated transcranial direct current stimulation and transcranial electric stimulation locations, maximizing reproducibility and participant comfort. The custom headgear with fixed electrode sites and a universal, one-size-fits-all design makes for a simplistic setup for 1×1 stimulation.

#### CBTp

The intervention consists of 16 individual-based weekly sessions, with follow-up assessments (1 month and 3 months after the intervention). The trial intervention is summarized in Table 1.

**Table 1:** Trial intervention.

Arms	Assigned intervention
Experimental: tACS^a^/CBTp^b^ (both active)	tACS: gamma band electric stimulation for 20 minutes at the beginning of each CBTp session. After 20 minutes, the device remains in place, but the electric stimulation will be automatically stopped. The CBTp will continue up to a total of 50 minutes/session. CBTp: 16 individual-based, weekly sessions each of 50 minutes in duration and 2× booster sessions at months 1 and 3 after intervention.
Sham comparator: sham tACS/CBTp (only CBTp active)	For the sham group, the investigators will use the same tES^c^ device; the sham stimulation will be applied only for a few seconds in the sham group. The CBTp will follow the same protocol for both arms.

^a^tACS: transcranial alternating current stimulation.

^b^CBTp: cognitive behavioral therapy for psychosis.

^c^tES: transcranial electric stimulation.

### Concomitant Medications/Interventions

#### Permitted/Supportive

Participants will be on their usual medication regimen, with no significant changes for at least 1 month (preferably 3 months) before enrollment. Small, symptomatic medication changes are permitted at the principal investigator’s judgment. These changes should not interfere with the assessments. For example, the use of benzodiazepines will be on hold from midnight of the day before the evaluations. Aside from antipsychotic medication, any pharmacological products are permitted as long as they are part of the usual medication regimen of the participant. Minor adjustments or symptom-treating medication (eg, insomnia and anxiety) are permitted at the principal investigator’s best judgment, provided they do not interfere with project activities.

Other interventions are permitted, but only if they are continuous and started 3 months before enrollment. For example, the participants would not be allowed to start a keto diet during the study, but they will be allowed to follow that diet if they started at least 3 months before enrollment, and it will be ongoing until the end of the study.

#### Not Permitted Concomitant Medications/Interventions

Participants must withhold benzodiazepines and other medications for sleep or anxiety for at least 8 hours before assessments, avoid using illicit drugs during research activities, refrain from hormone therapy throughout trial participation, and not engage in other CBTp or formalized psychosocial interventions concurrently with the study intervention.

### Participant Compliance

Being administered by professionals (and not self-administered), it is easy to appreciate the compliance with the intervention. The Personal Evaluation of Transitions in Treatment (PETiT) questionnaire assesses compliance with medication. Even if the participant agrees to participate in this study, they must continue to take their current pharmacological treatment.

Participants who are unable to follow instructions given to them about the study may be temporarily suspended or withdrawn from the study.

### Other Participant Care Considerations

The primary standard of care is medication management for adults with schizophrenia. When an individual requires other services or care, generally a referral is made to obtain these services. A proportion of adults with schizophrenia in the community may also have social or skill support via a community treatment team. The research laboratory is situated in a hospital setting, which is well resourced to support care in case of an emergency on-site. Participants will be receiving their standard, usual care throughout the study.

### Efficacy Assessments/Measurement of Effect

Therapeutic improvement, in our study, was defined as expected by statistically significant changes in some of our primary as well as secondary outcomes. To capture the progress in clinical improvement, we chose 2 validated scales that show high reliability and sensitivity to schizophrenia symptomatology—the PANSS and the NSA. The CFS was added as an additional measure for the executive function. We considered participants to be good responders to the intervention if there was an increase in PANSS General Scores higher than or equal to 20% of their baseline scores at the end of the intervention, with an improvement in scores of the PANSS Positive and PANSS Negative scales of at least 15% compared with the baseline. The therapeutic improvement also included a 20% decrease in scores for the NSA scale and a 20% increase in CFS scores at the end of the intervention, compared to baseline.

### Safety Considerations

An adverse event (AE) is any untoward medical occurrence in a patient administered an intervention (tACS in our specific project). An AE does not necessarily have a causal relationship with the treatment. An AE can be any unfavorable and unintended sign (including an abnormal finding or lack of expected interventional action), symptom, or disease temporally associated with the use of a tACS, whether or not related to tACS (definition based on the International Council for Harmonisation [ICH]). This includes any occurrence that is new in onset or aggravated in severity from the baseline condition, or abnormal results of any diagnostic procedures that are conducted within clinical practice.

An adverse intervention reaction (AIR) is defined as a response to an intervention (eg, tACS application) that is noxious and unintended. The phrase “response to an intervention” means that a causal relationship between the intervention and an AE is at least a reasonable possibility. The phrase “a reasonable possibility” means that there are facts, evidence, or arguments to support a causal association with the intervention. An AIR, in contrast to an AE, is characterized by the fact that a causal relationship between the intervention and the occurrence is suspected. All AEs judged by either the reporting physician or the sponsor as having a reasonable causal relationship to an intervention qualify as AIRs.

The AEs expected from tACS are a very mild tingling sensation, fatigue, a light itching skin sensation under the stimulus, a mild headache, and dizziness, all of which disappear just after stimulation. With tACS, no persistent AEs have been reported; however, the AEs associated with repetitive tACS application remain unclear. In this type of stimulation technique, no serious adverse effects were reported. Some questionnaires may ask about stressful events in their life, which may cause them to feel discomfort or recall past experiences that upset them. If they find the questionnaire content distressing, they are encouraged to inform the study coordinator immediately or at a later time.

A serious adverse event (SAE), based on ICH and EU Guidelines on Pharmacovigilance for Medicinal Products for Human Use, is any untoward medical occurrence that at any dose (with extending the meaning to the tACS intervention):

- Results in death if life-threatening (the patient was at risk of death at the time of the event; it does not refer to an event that hypothetically might have caused death if it were more severe);
- Requires inpatient hospitalization or prolongation of existing hospitalization;
- Results in persistent or significant disability/incapacity if a congenital anomaly/birth defect, if a suspected transmission of any infectious agent via a therapeutic intervention, or if medically important.

Medical and scientific judgment should be exercised in determining whether other situations warrant consideration as serious, such as significant medical events that, although not immediately life-threatening or resulting in death or hospitalization, could jeopardize the patient or necessitate intervention to prevent one of the aforementioned outcomes.

A product quality complaint (PQC) is defined as any suspicion of a product defect related to manufacturing, labeling, or packaging, that is, any dissatisfaction relative to the identity, quality, durability, reliability, or performance of a distributed interventional product, including its labeling, delivery system, or package integrity. A PQC may have an impact on the safety and efficacy of the product. In addition, it includes any technical complaints, defined as any complaint that indicates a potential quality issue during manufacturing, packaging, release testing, stability monitoring, dose preparation, storage, or distribution of the product or the drug delivery system. Upon identification of a PQC, investigators are encouraged to report it to the marketing authorization holder.

SAEs and AEs of interest (skin irritation and local burning sensation) are to be recorded in the participant’s source records. The collection of these events should start after the informed consent form is signed and will continue until the end of the observation period of the study. Safety evaluations will also include the collection of concomitant medications, as well as the collection of vitals. In addition, the suicidal ideation will be assessed using the Columbia Suicide Severity Rating Scale (C-SSRS) at baseline (exclusion criteria).

All SAEs assessed by the participating physician should be recorded in the participant’s source documents. For reports of hospitalization, it is the sign, symptom, or diagnosis which led to hospitalization that is the serious event for which details must be provided. Any event requiring hospitalization (or prolongation of hospitalization) that occurs during the study must be reported as an SAE, except hospitalizations for the following: (1) hospitalizations not intended to treat an acute illness or AE (eg, social reasons such as pending placement in a long-term care facility); (2) surgery or procedures planned before entry into the study (should be documented in the case report form [CRF]) [note: hospitalizations that were planned before the start of data collection, and where the underlying condition for which the hospitalization was planned has not worsened, will not be considered as SAEs]; (3) any AE that results in a prolongation of the originally planned hospitalization is to be reported as a new SAE; and (4) the cause of death of a participant in a study, whether the event is expected or associated with the intervention, is considered as an SAE.

### Pregnancy

Pregnancy here is no contraindication of tACS or CBTp during pregnancy. However, a participant in this special circumstance could have a different response to the intervention, influencing our final results. If a participant becomes pregnant during the study, data specified in the “data collection schedule” that is collected as part of the participant’s standard of care will continue to be recorded in the CRF for the applicable time points, but the participant will be withdrawn from the study.

### Statistical Considerations: Sample Size

This is a pilot randomized study with 28 participants (14 per arm). The primary goals are to evaluate recruitment feasibility, acceptability, and safety, and to produce unbiased estimates of the standard deviation and preliminary effect sizes of the primary outcome to inform sample-size calculation for a future definitive trial [31]. A target of 28 participants is consistent with common pilot rules-of-thumb (approximately 12-20 per arm) and provides sufficient precision to estimate recruitment and retention rates and the outcome variance. This pilot is not powered for hypothesis testing of efficacy (with n=14 per group, the minimal detectable standardized effect is large, ~*d*=1.1); outcome analyses will therefore be descriptive and will report effect estimates with 95% CIs as inputs for future sample-size calculations. To allow for expected attrition (approximately 15%-20%), we will recruit up to 33-40 participants. The generalizability of our findings is limited by the small sample size (n=28) and recruitment from a single treatment site. Patients in this cohort may differ from the broader schizophrenia population with respect to illness chronicity, symptom severity, comorbid psychiatric or medical conditions, and sociodemographic characteristics. In addition, the specialized treatment context may not reflect the experiences of individuals receiving care in community or primary care settings, where clinical resources and treatment approaches may vary. These factors restrict the extent to which the present results can be applied to other patient populations. Replication in larger, multisite, and more heterogeneous cohorts will be essential to establish the robustness and external validity of the findings.

The statistical analysis plan will use SPSS (version 29, IBM Corp) for the main tests, including effect size (Cohen *d*) and a 95% CI: repeated measures, mixed design analysis of variance, and *t* tests for two-group comparisons. Sex and gender differences will be examined using regression models that include biological sex, BSRI masculinity and femininity scores, and their interaction terms, with exploratory subgroup analyses based on BSRI classifications.

### Statistical Outcomes

#### Primary Outcome Measures

EEG gamma oscillations are fast neural rhythms linked to higher-order cognitive functions (attention, working memory, executive control, and consciousness). In the prefrontal cortex, gamma EEG activity is associated with working memory maintenance, cognitive flexibility, top-down attention control, and integrating information across cortical areas. Altered prefrontal gamma observed in schizophrenia. EEG measurement of gamma activity in the prefrontal cortex refers to detecting brain oscillations in the gamma frequency band (typically 30-100 Hz, often subdivided into low gamma: 30-50 Hz, and high gamma: 60-100 Hz) from scalp electrodes positioned over prefrontal areas. In a within-subject design, we will compare gamma power across time windows (pre- and postintervention). In a between-subject design, we use mixed models or *t* tests on summary metrics (mean power in time×freq window).

PANSS is a standardized clinical interview that rates the presence and severity of positive and negative symptoms and general psychopathology for people with schizophrenia within the past week [32]. The scale consists of 30 items: 7 positive, 7 negative, and 16 general psychopathology symptoms. The symptom severity for each item is rated according to a 7-point scale (1=absent to 7=extreme) to best describe the symptom’s presentation. The clinical interview takes approximately 45 minutes. The patient is rated from 1 to 7 on 30 different symptoms based on the discussion and reports of family members or primary care hospital workers. As 1 is the lowest score for each item, a patient cannot score lower than 30 for the total PANSS score. Scores are often given separately for positive items, negative items, and general psychopathology. [Time frame: PANSS will be applied at visit 1, screening (week 0); visit 4, end of intervention (week 17); visit 5, follow-up 1 (week 21); and visit 6, follow-up 2 (week 29).]

The NSA-16 assesses the presence, severity, and range of negative symptoms associated with schizophrenia [33]. For rapid clinical assessment or screening of patients for negative symptoms, the NSA-4 was developed as a simplified version, retaining only 4 of the 16 items: restricted speech quantity, reduced emotion, reduced social drive, and reduced interests. Each of the 4 items and the overall global negative symptoms is rated on a 1- to 6-point scale, where “1” represents no reduction from normal behaviors associated with the item and “6” represents a severe reduction in or absence of the behavior, with markedly impaired functionality. With respect to overall accuracy and predictive validity, the NSA-4 is comparable to the NSA-16. [Time frame: NSA-16 will be applied at visit 2, baseline (week 1); visit 4, end of intervention (week 17); visit 5, follow-up 1 (week 21); and visit 6, follow-up 2 (week 29).]

The CFS assesses cognitive flexibility as a factor that facilitates the individual’s adaptability toward events [34]. Cognitive flexibility refers to a person’s awareness of the existence of different communication styles in every situation, as well as their willingness and self-efficacy to employ these styles. The scale has 12 items pertaining to the 3 elements of cognitive flexibility: a person’s awareness of alternative communicative styles, willingness to be communicatively flexible, and self-efficacy in being communicatively flexible. The scale uses a 6-point Likert-type scale for each item (with 6 as “strongly agree,” 5 as “agree,” 4 as “slightly agree,” 3 as “slightly disagree,” 2 as “disagree,” and 1 as “strongly disagree”). The total score was used to calculate a global score ranging from 12 to 72, with questions 2, 9, 11, and 12 being reverse-coded. A higher score indicates higher cognitive flexibility. [Time frame: CFS will be applied at visit 2, baseline (week 1); visit 3, midpoint of intervention (week 9); visit 4, end of intervention (week 17); visit 5, follow-up 1 (week 21); and visit 6, follow-up 2 (week 29).]

#### Secondary Outcome Measures

HRV refers to the variation in time between consecutive heartbeats, a normal physiological phenomenon indicating the body’s ability to adapt to stress and demands. There is no single “ideal” HRV; a higher HRV generally indicates better cardiovascular health and fitness, with a normal range considered to be between 40 and 100 milliseconds. HRV provides insights into the body’s autonomic nervous system balance and overall psychological health. It reflects the variations in time intervals between heartbeats, indicating how well the body adapts to stress and recovers. However, individual HRV levels vary based on age, fitness level, and other factors, and tracking baseline HRV and trends for an individual is more important than comparing to others. [Time frame: HRV will be determined at visit 2, week 1, before and immediately after the first intervention (tACS/CBTp); it will also be determined pre- and post-16th intervention, visit 4, week 17.]

The World Health Organization Disability Assessment Schedule 2.0 (WHODAS 2.0) measures the level of functioning in 6 domains of life [35,36]: cognition—understanding and communicating; mobility—moving and getting around; self-care—attending to one’s hygiene, dressing, eating, and staying alone; getting along—interacting with other people; life activities—domestic responsibilities, leisure, work, and school; participation—joining in community activities and participating in society. The 36-item WHODAS 2.0 is the most detailed version (questions about the difficulties experienced in the past 30 days). It allows users to generate scores for the 6 functioning domains and calculate an overall functioning score. The average interview time for the interviewer-administered 36-item version is 20 minutes. The simple sum of the scores of the items across all domains constitutes a statistic that describes the degree of functional limitations. The scores range from 0 to 100, where higher scores indicate greater disability. [Time frame: WHODAS 2.0 will be performed at visit 2, baseline (week 1); visit 4, end of intervention (week 17); visit 5, follow-up 1 (week 21); and visit 6, follow-up 2 (week 29).]

The Quality of Life Enjoyment and Satisfaction Questionnaire—Short Form (Q-LES-Q-SF) is a 16-item, participant-scored survey used to quantify changes in quality of life [37]. The Q-LES-Q-SF evaluates general activities that are assessed in the longer form: physical health, feelings, work, household duties, school/course work, leisure time activities, and social relations. Participants rate their satisfaction using a 5-point scale ranging from 1 (very poor) to 5 (very good). A total score is derived from 14 items, with a maximum score of 70, higher scores indicating greater life satisfaction and enjoyment. [Time frame: Q-LES-Q-SF will be performed at visit 2, baseline (week 1); visit 3, midway intervention (week 9); visit 4, end of intervention (week 17); visit 5, follow-up 1 (week 21); and visit 6, follow-up 2 (week 29).]

The Brief Betrayal Trauma Survey is a semistructured interview that measures 14 potentially traumatic events involving mistreatment by someone close, not so close, and noninterpersonal events [38]. Respondents indicate how frequently they experienced an event before and after 18 years of age. It categorizes items into high, medium, and low betrayal. (Time frame: The Brief Betrayal Trauma Survey will be performed at visit 2, baseline.)

The Perceived Stress Scale (PSS) is one of the most widely used psychological instruments for measuring stress perception [39]. It measures the degree to which situations in one’s life are perceived as stressful. The 10 items inquire about how unpredictable, uncontrollable, and overloaded respondents find their lives, as well as the current levels of stress they experience. The questions in the PSS ask about feelings and thoughts concerning experiences during the last month. In each item, respondents are asked how often they felt a certain way. Individual scores on the PSS can range from 0 to 40, with higher scores indicating higher perceived stress. [Time frame: The PSS will be performed at visit 2, baseline (week 1); visit 4, end of intervention (week 17); visit 5, follow-up 1 (week 21); and visit 6, follow-up 2 (week 29).]

PETiT is a self-administered scale, which is user-friendly and sensitive to changes associated with treatment over time [26]. Consisting of 30 items, it assesses the respondent’s health over the last week and provides 1 of 3 responses (“often,” “sometimes,” or “never”). The scale assesses 2 highly relevant domains for schizophrenia: adherence-related attitude (includes 6 items reflecting adherence and feelings toward medication) and psychosocial functioning (24 items describing patient characteristics such as clarity, energy, concentration, functioning, sex drive, and memory). The PETiT total score ranges from 0 to 60, with higher scores indicating better patient health-related quality of life. [Time frame: PETiT will be performed at visit 2, baseline (week 1); visit 4, end of intervention (week 17); visit 5, follow-up 1 (week 21); and visit 6, follow-up 2 (week 29).]

The Working Alliance Inventory—Short Revised (WAI-SR), both forms, that is, for client and therapist, measures 3 critical aspects of the therapeutic alliance: agreement on the tasks of therapy, agreement on the goals of therapy, and development of an affective bond [40]. The 12 items are rated on a 5-point Likert scale ranging from “1=never” to “5=always.” The scores range from 5 to 20, with higher scores indicating a better therapeutic alliance. Completing the WAI-SR takes about 5 minutes. [Time frame: WAI-SR will be tested at visit 3, midterm through intervention (week 9); and visit 4, end of intervention (week 17).]

The BSRI is a psychological tool used to measure an individual’s perception of their own masculinity and femininity, and it is used to research gender roles, with the understanding that individuals can express both masculine and feminine traits. The interview has 12 items, with scores on a 1 (never or almost never true) to 7 (always or always true) scale, with an average calculated for masculine and feminine traits, and then categorizes them as masculine, feminine, androgynous, or undifferentiated based on those scores. Each item is assigned a category as follows: M for masculine, F for feminine, and N for neutral. [Time frame: BSRI is performed at visit 1, screening (week 0).]

The Stroop Color and Word Test (hereafter just Stroop test), developed by John Ridley Stroop, is a neuropsychological test that measures cognitive interference by asking participants to name the ink color of words, which are printed in a color that conflicts with the word itself. The Stroop scores are based on 3 factors—color naming, interference, and speed—and converted into a *T*-score for interpretation. A *T*-score of 40 or less is considered low, while a score above 40 is considered normal. [Time frame: The Stroop test will be applied at visit 2, baseline (week 0); at visit 4, end of intervention (week 17); and at the 2 follow-ups (week 21 and week 29).]

#### Other Prespecified Outcome Measures: Eligibility Screening Measures

The C-SSRS is a questionnaire developed by multiple institutions, including Columbia University, to help establish a person’s immediate risk of suicide [41]. Its questions are phrased for use in an interview format but can be completed as a self-report measure if necessary. The C-SSRS measures 4 constructs: the severity of ideation, the intensity of ideation, behavior, and lethality. It includes “stem questions,” which, if endorsed, prompt additional follow-up questions to obtain more information. The C-SSRS comprises 10 categories, all of which maintain binary responses (yes/no) to indicate the presence or absence of the behavior. The outcome of the C-SSRS is a numerical score with no specified clinical cutoffs due to the binary nature of the responses to items. Ultimately, interpretation is derived from a thorough clinical assessment, client history, and clinical expertise. [Time frame: at screening, visit 1 (week 0).]

The MoCA is a popular tool used in clinical practice when assessing for mild cognitive dysfunction. This instrument evaluates various cognitive domains, including attention, executive functions, memory, language, visuoconstructional skills, conceptual thinking, calculations, and orientation. Administering the MoCA takes around 10 minutes, and the total possible score is 30 points. A score of 26 or higher is considered normal, while a final score below 26 suggests mild cognitive impairment. [Time frame: MoCA will be performed at visit 1, screening (week 0), for participants over the age of 65 years (to exclude a possible cognitive decline).]

### Study Oversight and Data Management

Participant data will be collected as follows: electronic medical record review, wearable device data (EEG and HRV), electronic patient-reported outcomes (Stroop scores and BSRI) and patient-reported outcomes (in papers), paper clinician-administered scales and assessments, and study visit worksheets. The study will use a Microsoft Excel clinical database for electronic data capture. A change log will be maintained as an audit trail for this database.

Even with data security protections in place, there is a risk that participant information could be released by accident. In case of a confidentiality breach, despite the safeguards in place, the investigators will immediately take steps to contain it, notify the participant, investigate, and remediate. Only personnel with designated hospital appointments (Departmental Assistant Status or Departmental Research Assistant Status at Providence Care Hospital) or physicians at Providence Care Hospital who are part of the research team will have access to any personal health information. The access will be necessary to corroborate information given during interviewer-administered interviews linked with questionnaires. Access to medical records and/or study data will be limited to authorized personnel. Electronic data will be stored on a hospital or other institutional network with firewalls and other security and backup measures in place. Data stored on laptops or mobile devices will be encrypted. Paper copies of study data will be stored in locked filing cabinets in a secure location. The principal investigator and their delegate will have access to data. Data will be stored for 15 years under participant ID numbers (anonymized) on the electronic data, which will be stored on a hospital or other institutional network with firewalls and other security and backup measures in place. Any data that exists in an identifiable form (name) will be converted and replaced with participant ID immediately, at the first opportunity. A master list of code-linking identifiers will only be held by the principal investigator or delegate for 15 years after the end of the study. Data collected on paper, except the consent form, will be anonymized. Data collected on paper will be stored in the archive, in locked filing cabinets at Providence Care Hospital. The data will be kept for 15 years in a deidentified format in the archive. Data will be deposited on the Queen’s University Dataverse Collection in Borealis (a library-managed collection on a national platform).

Direct access to records will be provided to sponsor monitors as required and outlined in the study monitoring plan.

### Monitoring Plan

Monitoring will be performed on-site by the sponsor’s monitor in accordance with the study monitoring plan.

Trial monitoring is one of the principal quality control activities of the sponsor-investigator. Monitoring aims to ensure the participants’ rights, safety, and well-being, and the reliability of trial results as the trial progresses. Monitoring also verifies that the trial is conducted in compliance with the current research ethics board (REB)–approved protocol, TCPS 2 (Tri-Council Policy Statement 2), and ICH Guideline for Good Clinical Practice E6 and determines appropriate corrective and preventive actions when noncompliance is identified.

The sponsor-investigator has assigned a monitor that is not involved in the clinical conduct of the trial at the site and has completed training on this monitoring plan, the current versions of ICH Guideline for Good Clinical Practice E6 and TCPS 2, and the REB-approved protocol. All monitoring of this trial will be done on-site at Providence Care Hospital.

The monitor will assess investigator and study team qualification, training, and delegation of duties; trial master file and essential records (including standard of care); participant consent; appropriate and timely reporting of AEs, unanticipated problems, and SAEs; randomization process and maintenance of the blind; participant recruitment and retention rates (screening, enrollment logs, and withdrawals); data and device storage and security; device accountability, maintenance, and calibration; clinical trial data, including eligibility; protocol compliance, including endpoints, procedures, assessments, and interventions; source data verification; deviation or violation recording and reporting; and noncompliance and data trends.

The monitor will use standardized templates to review the above and document observations and findings. All observations will be discussed at the end of the on-site review with the sponsor-investigator and study team members. The monitor will review the observations, and all verified and confirmed findings are documented in a modular monitoring report. These reports will be provided to the sponsor and to the Queen’s Vice-Principal of Research Compliance and Training team to assist with addressing noncompliance and identification of trends. Sites will receive a letter outlining findings that require corrective and preventive action (CAPA). Monitors will work with the site to conduct a root cause analysis and formulate the CAPA.

The monitor will perform the first on-site review after the first participant has completed 2 weeks of treatment (study week 4). The second visit will be scheduled 5 weeks later, when the first patient has undergone the first set of on-treatment assessments. If no critical/significant or major findings or deviations have been identified, thereafter the monitor will conduct on-site reviews every 2 months while patients remain on active treatment (assuming a recruitment rate of >5 at the beginning of the study and lower thereafter). More frequent visits may be indicated for higher recruitment rates, rates of protocol deviations, or safety reporting.

As this study was assessed as being medium risk and will use monitoring tools from Network of Networks (N2), 100% source data verification of the following will be done for the first two participants randomized that receive intervention: informed consent process, eligibility, safety (AEs and SAEs), intervention data, endpoints (primary and secondary outcome measures), and protocol compliance. Propose thereafter an additional 25% (7/28) of participants and review for each baseline/eligibility at least one treatment period, end of study, one follow-up, and all endpoints or events (SAEs, death, etc). Additional sources of data verification and visits may be added based on significant error rates or noncompliance. The study monitoring plan will be reviewed annually to ensure that the processes and procedures outlined herein remain applicable to the conduct of the study. Additionally, if amendments are made to the protocol that affect the study procedures and/or participant safety, a review of the monitoring plan will be performed to revise the plan as needed.

### Ethical Considerations

This study adheres to the principles outlined in the Tri-Council Policy Statement (2022) and ICH Guideline for Good Clinical Practice E6. It complies with the REB guidelines, involving human participants, medical records, patient information, and observations of public behaviors. The study protocol, along with required updated templates and the letter of consent and information, was reviewed and approved by Queen’s University, Health Sciences Research Ethics Board (HSREB), file number PSYC-222-23/6038935/6044118 TRAQ DSS 6036290 (last approval from July 2, 2025), before any research activities commenced. All study participants receive the HSREB-approved detailed consent form, which explains the study and provides enough information for informed decision-making regarding their participation; consent for the involvement is obtained before any study procedures are conducted and is signed by the participant, the person who conducted the informed consent discussion, and the principal investigator. For secondary analyses using existing data with primary consent, the original consent HSREB approval covers secondary analysis without additional consent.

### Risks of Participation

There are risks to taking part in any clinical study. Risks associated with tACS are generally mild and disappear after the stimulation. With tACS, no persistent AEs have been reported; however, the AEs related to repetitive tACS application remain unclear. In this type of stimulation technique, no serious adverse effects were reported. Some of the side effects that may occur during this study can be treated. The AEs are a very mild tingling sensation, fatigue, a light itching sensation under the stimulus, mild headache, and dizziness, all of which disappear just after stimulation.

Participants in one of the two intervention groups will not receive the current stimulation but a placebo/sham tACS (the tACS is a placebo, but the CBTp is the intervention). Without receiving the low electric stimulation that may augment their response to psychotherapy (CBTp), their condition might not improve. The previous studies using only CBTp as an intervention reported improvement of the illness (they will receive CBTp anyway). However, these study participants will continue their usual treatments, including antipsychotic treatment.

While generally safe, CBTp can have some risks, primarily during initial sessions or specific techniques such as exposure therapy. These risks can include short-term stress, anxiety, or emotional distress while exploring painful feelings and experiences. There is limited scientific research on the possible side effects of psychotherapy, including CBTp. The risks of CBTp are generally low and can be managed with open communication and the support of a skilled therapist.

Some questionnaires used in this study may ask about stressful events in a participant’s life, which may cause them to feel discomfort or recall past experiences that may have upset them. If they find the questionnaire content distressing, they are encouraged to inform the study coordinator immediately or at a later time. A member of the research team can discuss this with them and refer this to a member of their care team whom they identify and consent to (the investigators will ask them for written consent to disclose this information if they decide). The participant’s name will never be placed on a questionnaire to maintain their privacy and protect their confidentiality. If they write their name by accident, a sticker containing their participant ID will be placed over it to preserve their privacy. The participants may also feel frustrated or confused by the language in the questionnaires. If investigators need clarification on any points in the written material or explanations, the research team will be happy to assist and provide further details to their satisfaction.

The research team will manage the plan to mitigate psychological and emotional risks. To achieve this, interviewers will be trained to administer questionnaires thoughtfully, sensitively, and with awareness of the types of questions being asked, as well as the potential effects and thought processes participants may experience. These are essential strategies to ensure sensitive and polite communication, helping researchers learn to interpret verbal and nonverbal cues that may convey discomfort.

### Privacy and Confidentiality

Participant names will remain confidential and not be disclosed to those not involved in the study. In the CRF, only the participant number will be recorded. If any identifiers appear on any document, they will be redacted in copies stored in the site master file or presented for audit. Data stored electronically will comply with local data protection laws. Participants will be informed that ethics committees or research ethics boards may inspect their records and that all personal information will be treated with utmost confidentiality in line with local data protection laws. The investigator will keep a list linking participant numbers to names for record identification and retrieval.

EEG sample recordings will be stored in a file on the designated laptop, where they will remain anonymous, identified only by a code number. Postrecording, the samples will be transferred to a secure server at Queen’s University, anonymized, and then deleted from the original recorder. These anonymized files will be stored on the server for 15 years after data collection ends, after which they will be permanently deleted.

Any chart reviews will be conducted by hospital-appointed individuals who have the privilege to access them.

## Results

### Duration of Participation

The timeline for recruitment, treatment, and follow-up is 18 months, followed by 6 months for data analysis, writing manuscripts, and dissemination activities.

Total duration of participation in this research will be 29 weeks; the visits, procedures, and timelines are represented in Table 2. In addition to the study visits, participants will be seen weekly for 16 weeks to receive CBTp with either tACS or sham tACS, and there will be a booster session intervention during each of the two follow-up visits.

**Table 2:** Study calendar, visits, procedures, and timelines^a^.

Study phases	Phase I: Preintervention		Phase II: Intervention		Phase III: Postintervention	
Timeline	Visit 1	Visit 2	Visit 3	Visit 4	Visit 5	Visit 6
Procedure	Screening	Baseline	Session 8	Session 16	Follow-up	Follow-up
Duration	Week 0	Week 1	Week 9	Week 17	Week 21	Week 29
Informed consent, 60 minutes	✓					
Chart review, 60 minutes	✓					
Height and weight, 10 minutes	✓					
Vital signs, 15 minutes	✓	✓	✓	✓	✓	✓
Physical exam, 20 minutes	✓					
MoCA^b^/age >65 years, 20 minutes	✓					
C-SSRS^c^, 10 minutes	✓					
Bem Sex-Role Inventory, 15 minutes	✓					
Chart review, 30 minutes	✓					
Physical exam, 20 minutes	✓					
Vitals, 10 minutes	✓	✓	✓	✓	✓	✓
PANSS^d^, 40 minutes	✓			✓	✓	✓
NSA-16^e^, 15 minutes		✓		✓	✓	✓
WHODAS 2.0^f^, 10 minutes		✓		✓	✓	✓
CFS^g^ and PSS^h^, 10 minutes		✓	✓	✓	✓	✓
PETiT^i^, 10 minutes		✓	✓	✓	✓	✓
Q-LES-Q-SF^j^, 5 minutes		✓	✓	✓	✓	✓
BBTS^k^, 10 minutes		✓				
WAI-SR^l^, 5 minutes			✓	✓		
Stroop^m^, 10 minutes		✓		✓	✓	✓
EEG^n^, 30 minutes including prep.	✓			✓		
HRV^o^ (beginning and end of session), 5 minutes		✓		✓		

^a^Benzodiazepines and other medications for sleep or anxiety must be held for at least 8 hours before assessments. This questionnaire/assessment must be done at approximately the same time of day each time. If these cannot be done during a scheduled visit, the participant may be rescheduled for another day. Participants will be allowed to take short breaks during these assessments if they need them.

^b^MoCA: Montreal Cognitive Assessment.

^c^C-SSRS: Columbia Suicide Severity Rating Scale.

^d^PANSS: Positive and Negative Syndrome Scale.

^e^NSA-16: 16-item Negative Symptom Assessment.

^f^WHODAS 2.0: World Health Organization Disability Assessment Schedule 2.0.

^g^CFS: Cognitive Flexibility Scale.

^h^PSS: Perceived Stress Scale.

^i^PETiT: Personal Evaluation of Transitions in Treatment.

^j^Q-LES-Q-SF: Quality of Life Enjoyment and Satisfaction Questionnaire—Short Form.

^k^BBTS: Brief Betrayal Trauma Survey.

^l^WAI-SR: Working Alliance Inventory—Short Revised.

^m^Stroop: Stroop Color and Word Test.

^n^EEG: electroencephalogram.

^o^HRV: heart rate variability.

### Current State of the Research

The enrollments for this pilot clinical trial are in September 2025. By November 1, 2025, we have enrolled 15 participants. Two of them were screening failures, and one withdrew from the study after intervention session 2—the reasons for withdrawal were not related to our research (they started a full-time job and were no longer able to attend the weekly sessions). The 12 participants who remained in the study are following the intervention protocol, with 8 receiving both active tACS/CBTp and 4 receiving sham tACS/CBTp. The gender distribution of the screening process so far is 7 females, 4 males, and 1 transgender female, with 7 of them under the age of 40 years and 7 older than that. Two male participants were screened for failure, and one female left the study.

## Discussion

### Expectations

Based on well-established evidence of cognitive deficits in schizophrenia, as well as our own observations in the CBTp Lab, an additional weakness in current schizophrenia treatment protocols was identified. Aside from challenges in therapeutic engagement, patients struggle to retain progress made in individual sessions. This is because the cognitive model-based approach requires implementing actual cognitive and behavioral changes—the “action plan/homework”—without which the therapeutic efforts of both the therapist and patient become futile. Current guidelines, suggesting an hour-long session of CBTp weekly for at least 16 weeks, become insufficient in the context of a complex symptomatology imbued with negative symptoms. A need for a much more concerted effort to obtain significant improvement becomes clear if we were to close the gap of disadvantages that schizophrenia patients are confronted with.

This study hypothesizes that tACS applied at the beginning of the CBTp intervention in people with schizophrenia will predict better clinical and functional responses to therapy (compared with the sham tACS/CBTp).

The expectations are that CBTp will be more efficient in the participants undergoing active tACS compared with sham tACS. Considering that cognitive and emotional status are gender dependent (hormonal differences, brain structure, and sociocultural influences), we expect that the therapeutic response could be gender specific [42,43].

The impact of this research is represented by clinical and social benefits, contributions to the scientific literature, and support for new research applications.

### Potential Clinical Benefit

A total of 28 clients will have the opportunity to benefit from our CBTp intervention with or without tACS.

### Social Benefits

The new type of intervention will allow for personalized and highly impactful treatment. This will lead to quality-of-life improvement for participants as well as for support members (family and caregivers) and will reduce health care costs.

### Disseminations

Aside from the current published protocol and the first scoping review on this topic [18], the investigators anticipate publishing up to 5 new peer-reviewed publications and presenting the results at different scientific events. Two graduate students are team members of this research, and by the end of 2026, they will write their master's theses from the preliminary data of this clinical trial. This pilot study aims to test and redefine our research methods, procedures, and feasibility of the main study. Based on the preliminary/interim analysis, we intend to apply for funding to further expand on the correlation between specific frequency brain stimulation and clinical outcomes in schizophrenia. Adding brain imaging evidence will help us to prove the efficacy of our new intervention protocol.

### Limitations

Limitations to the pilot studies include small sample sizes, resulting in less generalizable results. To our knowledge, this intervention protocol, tACS/CBTp, has never been used before in people living with schizophrenia. This study would not only be the first of its kind to highlight the potential capabilities of this therapy (associated with regular antipsychotics) in treating cognitive and negative symptoms, but also to expand the scope of pre-existing literature surrounding the augmentation of the CBTp efficacy.

### Further Directions

Replication of this research protocol with a larger sample (N≥30) would allow for greater specificity and rigor in distinguishing predictors, suggesting clear selection criteria for tACS/CBTp candidates.

### Conclusions

This study will help us develop a new interventional protocol that could impact the outcome of schizophrenia at personal, familial, and social levels, reducing the rate of relapse and the costs of hospitalization.

## Abbreviations

AE: adverse event
AIR: adverse intervention reaction
BSRI: Bem Sex-Role Inventory
CAPA: corrective and preventive action
CBTp: cognitive behavioral therapy for psychosis
CFS: Cognitive Flexibility Scale
CRF: case report form
C-SSRS: Columbia Suicide Severity Rating Scale
DSM-5: Diagnostic and Statistical Manual of Mental Disorders (Fifth Edition)
EEG: electroencephalogram
HRV: heart rate variability
HSREB: Health Sciences Research Ethics Board
ICH: International Council for Harmonisation
MoCA: Montreal Cognitive Assessment
NSA-16: 16-item Negative Symptom Assessment
PANSS: Positive and Negative Syndrome Scale
PETiT: Personal Evaluation of Transitions in Treatment
PQC: product quality complaint
PSS: Perceived Stress Scale
Q-LES-Q-SF: Quality of Life Enjoyment and Satisfaction Questionnaire—Short Form
REB: research ethics board
SAE: serious adverse event
tACS: transcranial alternating current stimulation
TCPS 2: Tri-Council Policy Statement 2
WAI-SR: Working Alliance Inventory—Short Revised
WHODAS 2.0: World Health Organization Disability Assessment Schedule 2.0
